# Health risk of consuming *Sphoeroides* spp. from the Navachiste Lagoon complex due to its trace metals and organochlorine pesticides content

**DOI:** 10.1038/s41598-022-22757-1

**Published:** 2022-11-01

**Authors:** Gabriela Muñoz-Armenta, Ernestina Pérez-González, Guadalupe Durga Rodríguez-Meza, Hector Abelardo González-Ocampo

**Affiliations:** Instituto Politécnico Nacional, CIIDIR-UNIDAD SINALOA, Blvd. Juan de Dios Batiz Paredes #250, 81101 Guasave, SIN Mexico

**Keywords:** Environmental impact, Marine chemistry

## Abstract

The Navachiste complex (NAV) is impacted by neighbored human activities and is located in the southwestern coastal zone of the Gulf of California. The study determines the trace metal (TM) and organochlorine pesticides (OCP) health risk content in the edible tissue of *Sphoeroides* spp. from NAV. The daily intakes (EDI), target hazard quotient (THQ), hazard index (HI), and carcinogenic and non-carcinogenic risks were calculated. Twenty OCP and seven TM were detected. Cd, Cu, Fe, Mn, Pb, and Zn were above MRLs. The γ‒Chlordane was the most frequent OCP. The highest average concentration was for α‒HCH, followed by γ‒chlordane. With the high ratios of γ‒HCH, p, p′‒ DDD and p, p′‒DDD, and the absence of p, p′‒ DDT, the higher ratios for dieldrin and endrin than for aldrin, α‒ chlordane, γ‒chlordane, heptachlor, and heptachlor epoxide indicates historical contamination. In contrast, the residual products of methoxychlor, endosulfan, and its isomers indicate endosulfan's recent use. The TM EDI, THQ > 1 (at 120 g day^−1^), and the ILCR (> 1 × 10^–6^) were above minimum levels, showing a high-risk potential for cancer development in the long term.

## Introduction

Seafood consumption by humans contributes significantly to the intake of eicosapentaenoic (EPA) and docosahexaenoic (DHA) acids, vitamin D, and other micronutrients that are essential to keep a healthy life^1,2^. Despite knowing the origin of these micronutrients, consumers usually show no preference for certain seafood products, certification, or where the seafood was captured^3^, and some of these organisms could contain toxic substances above maximum residue levels (MRLs) that might risk people's health^4^. Food safety is essential to prevent these health risks regarding toxic pollutants in seafood caused by anthropogenic activities^5^. Among the most toxic anthropogenic pollutants are organochlorine pesticides (OCPs) and trace metals that are environmentally persistent, become bioaccumulated and biomagnified due to their low water solubility and slow chemical decomposition^6^. The OCPs and trace metal residues are transported to the marine ecosystems by environmental factors such as effluents, wind, and rain, becoming bioavailable from marine sediments or water columns for the marine organisms^7,8^.

The persistence of trace metals and OCPs in the environment allows them to be found as residues in areas far away from the original sites of their application^9^. Trace metals, such as Pb and Cd, are elements that interfere in biochemical metabolic processes^10^. Other trace metals such as Fe, Zn, and Cu, when they are above MRLs, might cause health problems inhibiting nutrient absorption^11^.

The persistence of these toxic pollutants makes their study crucial to determine their health impact as a human health concern^12^.

**Figure 1:**
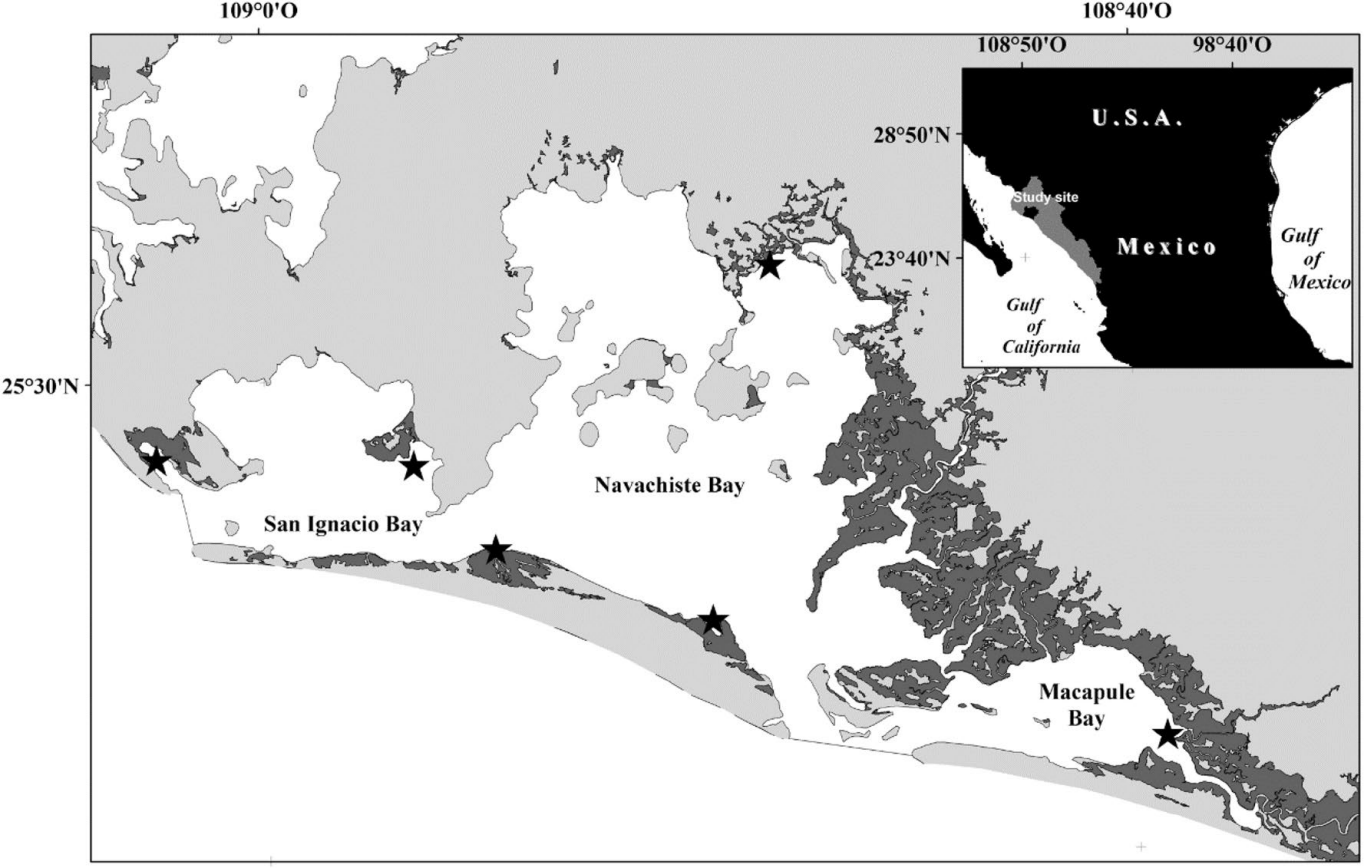
Navachiste coastal lagoon complex location and sampling collection sites (black stars) of fillet of *Sphoeroides* spp. for analysis of their trace metal content. Map was constructed using the ARCGIS DESKTOP® Ver. 10.8.2 with the authorization number ESU 125678848 using the georeferenced shapefiles the ARCGIS®^14^ and CONABIO, Mexico^15^ databases, field data converted to grid data.

The present study was performed in the Navachiste complex coastal lagoon (NAV), which is constantly impacted by the inputs of the wastewaters of the adjacent agricultural Guasave Valley, low-density tourism, urban and aquaculture activities. The NAV lagoon complex encompasses three coastal lagoons that geologically include coastal plains (55%), with dunes and salt flats (4.3%), deltaic depositional facies (21%), marshes and salt flats (17%), beach and sand bars (2%), and low mountains of steep slopes with dunes (0.5%)^13^ (Fig. 1). The field data was converted to grid data, and spatial analysis was performed with the ARCGIS DESKTOP® Ver. 10.8.2 with the authorization number ESU 125678848 using the georeferenced shapefiles from the ARCGIS®^14^ and CONABIO, Mexico^15^ databases (Supplementary table 1). Previous studies in the area have reported the presence of the OCPs and trace metals in edible tissues of inhabiting seafood species^16–20^. Among the polluted seafood species reported in the NAV are those of the Tetraodontidae family, like the pufferfish *Sphoeroides* spp.^21^, a euryhaline fish that inhabits coastal lagoons and estuaries^22^, and its feeding habits include zoo benthivores and omnivores, including bivalves, gastropods, and macrophytes^23–25^. The commercial value of the pufferfish *Sphoeroides* spp. is sizable and its catch reached, in Sinaloa in 2020, more than 708 tons with a value of more than 3 million USD, and its capture has been increasing in the last decade^26,27^. However, due to the constant pollution with OCPs and trace metal residues of the NAV, the risk of being subjected to carcinogenic or non-carcinogenic effects by the consumption of *Sphoeroides* spp. is evident. In this sense, the aim of the present study was to evaluate the carcinogenic and non-carcinogenic risk of the *Sphoeroides* spp. from the NAV.

## Methods

Eighty-six fillet of the pufferfish *Sphoeroides* spp. were obtained from the local fishers with a fishery permit in the NAV, located in the southern part of the Gulf of California in Mexico between 25.4° and 25.7° N and 108.85°–108.55° W^28,29^. None of the specimens were taken alive to perform the following laboratory experiments that were performed in accordance to national or international agreements guidelines and regulations.

Pufferfishes were obtained once every quarter of a year between summer (July 2016) and autumn (October 2017). Sampling points were selected among frequent fishing areas identified by local fishers. Frozen pufferfishes were dissected to remove the fillet that was divided into two parts, one part was kept in aluminum foil for posterior OCP content analysis, and the other part was kept in Ziploc® bags for trace metal content analysis. All samples were kept frozen in an ice acid-based cooler and transported for storage in a freezer in the Pollution laboratory located in the CIIDIR-SINALOA, Mexico. During each sampling period, pH, temperature (°C), salinity (‰), and dissolved oxygen (DO) were registered with a multiparameter HANNA® HI-9828 (HANNA Instruments, Woonsocket, RI, USA).

### Trace metal analysis

Acid digestion with nitric acid according to Méndez et al.^30^ was the procedure used for the trace metals extraction from muscle, and determined by atomic absorption spectroscopy. Each collected *Sphoeroides* spp. sample was dehydrated until constant weight. Five grams of dehydrated sample was digested in Erlenmeyer flasks with a concentrated HNO_3_ and HCL acids (1:5) mixture. Samples in the digestion procedure were boiled on a heating plate until dissolved. Cooled samples were mixed in 24 mL of deionized water and 1 mL of HCl, and taken to 50 mL with deionized water, and stored at room temperature for later analysis. The trace metals were determined by atomic absorption spectrophotometry (AVANTA GBC ®, US) with an air/acetylene flame burner and hollow cathode lamps. Trace metal standards of 0.125, 0.25, 0.5, 1, 2, and 4 ppm were prepared to verify the accuracy of the instrument. The quality control of the procedure was achieved using PACS and MESS reference materials (Supplementary table 2) in blank samples which were analyzed after each 10 of digested samples.

### Organochlorine pesticides analysis

The extraction, purification, analysis and quantification of OCP in the fillet samples were performed following the modified EPA-8081b^31^. Was macerated 10 g of sample with 5 g of Na_2_SO_4_ (Sigma Aldrich, St. Louis, MO, US) and 30 mL of hexane (chromatographic grade). The recovered hexane was purified with a clean-up column (fiberglass wool, alumina, florisil, silica gel, and anhydrous sodium sulfate, at a proportion of 2:1:1:1:3, respectively). The extracts were concentrated and completely dried in a hood, and then dissolved in 2 mL of isooctane.

The gas chromatograph (Perkin Elmer® XL, Auto System®, Perkin Elmer, Inc., Waltham, MA, US) is coupled to a ^63^Ni-ECD detector, TotalChrom Navigator® software, and DB-5 column (Agilent®). A six-point calibration curve was constructed (0.001–0.05 µL mL^−1^). The chromatography conditions were: oven heating ramp to 120 °C (for 1 min) until 240 °C increasing at a rate of 4 °C min^−1^; EDC at 300 °C; injector at 260 °C; 2 µL of sample injection; split-split less on; attenuation of 16; nitrogen as a carrier gas at 8.7 psi at 30 mL min^−1^. Pesticide mix standards (EPA 8081®) and pesticide subrogate mix (SUPELCO® Cat. CRM46845 and CRM48460, respectively), and linearity, detection limit, and recovery range (Supplementary table 3) were calculated to ensure the accuracy of the results.

A database was constructed with Microsoft Excel®, and data were statistically analyzed with Minitab17® software. A Kolmogorov Smirnoff normality test, ANOVA, and posthoc Tukey test were performed to determine significant differences.

### Human health risk assessment

Estimated daily intakes (EDI) for trace metals (TM) and OCP in the *Sphoeroides* spp. fillet from the NAV were calculated^8,32,33^ (Eqs. 1, 2):

1 $${EDI}_{TM}= \frac{{TM}_{C }\times {VIR}_{d}\times ED\times EF}{BW \times AT}$$

2 $${EDI}_{OCP}= \frac{{VIR}_{d}\times {OCP}_{C}}{BW}$$

where, *TM*_*C*_ or *OCP*_*C*_ = average TM (μg g^−1^) or OCP (mg kg^−1^) concentration in fish tissue (dry weight); *VIR*_*d*_ = average daily fish consumed in Mexico (32.88 g day^−1^), *ED* = exposure duration (26 years), *EF* = exposure frequency (365 days year^−1^), *BW* = Mexican adult average human weight (74.3 kg), and *AT* = average time (365 days year^−1^ per 26 years).

### Non-carcinogenic risk

For TM the target hazard quotient (THQ) (Eq. 3) and the hazard index (HI) (Eq. 4)^32,34,35^ were used to calculate the non-carcinogenic risk.

3 $${THQ}_{noncancer}= \left(\frac{EF\times ED \times {VIR}_{d} \times {TM}_{C}}{RfD+ BW+ {AT}_{noncancer}}\right) \times {10}^{-3}$$

4 $$HI= \sum_{n}^{i}{THQ}_{n}$$

If THQ or HQ is below 1 represents non-carcinogenic risk; if it is above 1 non-carcinogenic risk can occur.

For OCP*,* the non-carcinogenic risk was assessed by the hazard quotient (HQ) (Eq. 5) and the total hazard quotient (THQ)^36,37^ (Eq. 6):

5 $$\text{HQ }= \text{ } \frac{EDI}{{RfD}_{OCPx}}\times 100$$

6 $$THQ= \sum_{i=1}^{n}{HQ}_{i}$$

where, *RfD*_*OCPx*_ = reference doses for “x” OCP.

HQ or THQ values lower than 100 represent a non-carcinogenic risk, but HQ or THQ above 100 means that non-carcinogenic symptoms can occur.

### Carcinogenic risk

For the TM the risk of cancer for each TM was calculated by the incremental lifetime cancer risk (ILCR)^38^ (Eq. 7):

7 $$ILCR={EDI}_{TMx}\times {CSf}_{TMx}$$

*CSf* = Cancer slope factor of carcinogenic TM estimated^39^.

For OCP the carcinogenic risk (*CR*_*lim*_) was calculated^40^ (Eq. 8):

8 $${\text{CR}}_{{{\text{lim}}}} { =}\frac{{{{{\text ARL}~ \times ~{\text BW}}}}}{{\mathop \sum \nolimits_{{m = 1}}^{x} C_{m} ~ \times ~CSF_{{OCPx}} }}$$

where *CRl*_*im*_ is the maximum allowable consumption rate for an aquatic product (kg day^−1^); *ARL* is the maximum acceptable individual lifetime risk level, which is dimensionless and a value of 10^–5^ was used in this study^41,42^; *CSF*_*OCPx*_ is the cancer slope factor of *OCP*_*x*_ for a carcinogenic risk (mg kg^−1^ day^−1^).

## Results

### Morphometric data and TM and OCP concentrations in fish edible fillet

In this study, 20 OCP and seven trace metals (TM) were detected in the *Sphoeroides* spp. fillets from NAV. Some TM concentrations were detected above MRLs and showed a carcinogenic and non-carcinogenic risk for human consumption. A not normal distribution was found with the Kolmorov-Smirnoff (KW) test (α = 0.05), concentration of trace metals (KS = 0.117, *p* < 0.01), size (KS = 0.141, *p* = 0.010), weight (KS = 0.211, *p* < 0.010), and OCPs concentration (KS = 0.43, *p* < 0.01) (Table 1). The average length and weight of specimens (24.028 cm and 334.28 g, respectively), were similar to specimens of the same genus from shallow brackish water areas^43^. A correlation was found between weight (Pearson, α = 0.05, *p* = 0.001) and height (Pearson, α = 0.05 *p* = 0.0005) and with the concentration of trace metals (Pearson, α = 0.05 *p* = 0.179).

**Table 1:** Frequency percentage, mean concentration, standard deviation (± SD) of trace metals and OCPs in the fillet of *Sphoeroides* spp. from NAV, Mexico.

Trace metal	Frequency n = 86	Concentration^1^	± SD	MRLs
Cd	1.16	1.45	0	3 × 10^−5ai^–0.002^a^
Cu	41.86	**5.06**	21.06	0.03–0.12^b^
Fe	100	**80.52**	73.54	0.7–0.8^c^
Mn	38.37	**6.13**	3.86	1^ab^
Ni	32.56	8.06	3.53	0.0005^a^–0.14^b^
Pb	83.72	**18.42**	16.53	0.0003^ai^–0.004^a^
Zn	100	**189.55**	1062.73	0.03–0.12^ab^

OCPs				
Aldrin	3.49	0.80	0.93	10^d^
*p, p'‒*DDD	10.47	0.11	0.07	1250^e^
*p, p'‒*DDE	26.74	0.12	0.19	1250^e^
*p, p'‒*DDT	2.33	ND	ND	1250^e^
Dieldrin	15.12	0.13	0.23	10^d^–100^h^
Endosulfan I	32.56	0.25	0.38	50
Endosulfan II	22.09	0.21	0.16	50
Endosulfan sulfate	11.63	4.93	9.85	50
Endrin	16.28	2.22	4.94	10^d^–50^h^
Endrin aldehyde	33.72	0.05	0.09	10^d^
Endrin ketone	13.95	4.93	6.96	50^h^
Heptachlor	1.16	0.17	0	10^d^–20^e^
Heptachlor epoxide	3.49	0.07	2.02 × 10^–6^	10^d^
Methoxychlor	18.6	6.14	22.8	10^f^
α‒HCH	12.79	24.7	32.3	10–250^e^
β‒HCH	6.98	3.52	7.14	10–250^e^
γ‒HCH	1.16	1.71		5–250^e^
δ‒HCH	10.47	5.33	13.2	250^e^
α‒Chlordane	3.49	0.02	0	2–50^g^
γ‒Chlordane	60.47	22.94	72.5	NE

Bold numbers are pollutants concentration above MRLs; ND Below limit of detection.

^1^Concentration is for TM mg kg-1 dry weight; OCPs μg kg-1 wet weight.

^a^NSSP, 2019;^b^Anandkumar, et al., 2019;^c^Selvam, et al., 2021;^d^Adeleye, et al., 2019;^e^Baqar, et al., 2018;^f^Buah-Kwofie, et al., 2019;^g^Oyekunle, et al. 2017;^h^Chandra, et al., 2021;^i^Rajkowska-Myśliwiec, et al., 2022.

### TM concentration in *Sphoeroides* spp.

The TM concentrations in *Sphoeroides* spp. presented the following trend: Zn > Fe > Pb > Ni > Mn > Cu > Cd, from which, Cd, Cu, Fe, Mn, Pb, and Zn were above MRLs established by some countries´ Environmental Protection Agencies^44–51^, or were higher than in other marine fish^52–55^. Cd was detected in just one specimen.

In this study, the average concentrations of Cd (1.45 mg kg^−1^ dw) was above the MRLs (3 × 10^–5^–2 × 10^–3^ mg kg^−1^ dw). Cu average concentration (1.45 mg kg^−1^ dry wt) showed values above MRL (0.03–0.12 mg kg^−1^ dw). Zn was detected in 100% of samples, with an average concentration (189.55 mg kg^−1^ dw) higher than the recommended MRLs (0.03–0.12 mg kg^−1^ dw). The average concentration of Fe detected in this study (80.52 mg kg^−1^ dry wt) was below the MRL (0.7–0.8 mg kg^−1^ dw). The Pb concentration was the third highest in the samples analyzed, and the average concentration for Pb (18.42 mg kg^−1^ dw) exceeded the recommended MRL (0.0003–0.004 mg kg^−1^ dw). Ni was detected in a third of the samples (32.56%) with an average concentration (8.06 mg kg^−1^ dw) below the MRLs (0.0005–0.14 mg kg^−1^ dw). The average concentration of Mn (6.13 mg kg^−1^ dw) detected in 38.37% of the samples was above the MRLs (1 mg kg^−1^ dw) (Table 1).

### OCP concentrations in *Sphoeroide*s spp.

Twenty-two OCPs were detected in the muscle of *Sphoeroides* spp., several of them already listed as prohibited by the member countries of the WTO^56^. γ‒Chlordane was the most frequent OCP, and the analytes with the highest average concentration were α‒HCH, followed by γ‒chlordane (Table 1). No relation was found between size and OCPs concentration (Kruskal–Wallis, α 0.05, *p* = 0.442), nor between weight and pesticide concentration (Kruskal–Wallis, α 0.05, *p* = 0.438). Others studies in fish, the concentration of OCPs in tissues follows the following order of magnitude: liver > intestine > skin > muscle^57,58^, but, in the present study, the presence of these contaminants was determined only in the muscle of *Sphoeroides* spp. to assess their risk due to consumption.

Among the OCPs determined in the muscle of *Sphoeroides* spp. were HCHs, such as *α*‒HCH (24.7 µg kg^−1^ ww), *β*‒HCH (3.52 µg kg^−1^ ww), *γ*‒HCH (5.33 µg kg^−1^ ww), and δ‒HCH (3.52E-03 mg kg^−1^ ww), none of these concentrations were above MRLs (Table 1).

The DDT was detected in only two samples below the detection limit, but isomers, *p, p'*‒DDE (0.12 µg kg^−1^ ww) and *p, p'*‒DDD (10.11 µg kg^−1^ ww) were detected with a frequency of 26.74 and 10.47%, respectively.

From the drin family, aldrin (0.8 µg kg^−1^ ww), dieldrin (0.13 µg kg^−1^ ww), and endrin (2.22 µg kg^−1^ ww) were detected, with a frequency of 3.49, 15.12 and 16.28%, respectively. None of these OCPs were detected above MRLs.

Chlordane for technical use consists of a mixture of the stereoisomers *α*‒chlordane, *γ*‒chlordane, heptachlor, and heptachlor epoxide, which present concentrations of 0.02, 22.94, 0.17, and 0.07 µg kg^−1^, and a frequency of 60.47, 3.49, 1.16 and 3.49%, respectively. In the present study, the detected concentrations of these OCPs were lower than the MRLs (Table 1).

The endosulfan technical product consists of 70% endosulfan I and 30% endosulfan II, whose concentrations (0.25 and 0.21 µg kg^−1^, respectively) were below the MLRs (Table 1). Methoxychlor at a mean concentration of 6.14 µg kg^−1^ ww, was detected in 18.6% of the samples, and the concentration detected is within the permissible limits in Mexico (Table 1).

### Seasonal concentrations

**Figure 2:**
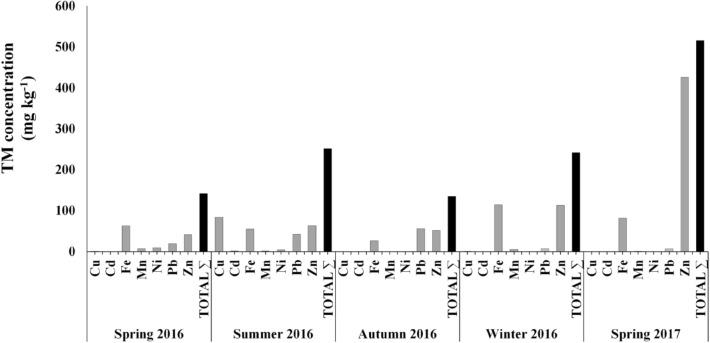
Seasonal concentration of TM in the *Sphoeroides* spp. fillet from Navachiste coastal lagoon.

The highest TM concentration was detected in the spring of 2017, when Zn and Fe were at the highest average concentration (425 and 81.96 mg kg^−1^). The spring and summer of 2016 showed the highest diversity of TM, whereas the autumn 2016 and spring 2017 had the lowest diversity. Most average TM concentrations among the seasons depicted values below 90 mg kg^−1^ (Fig. 2).

**Figure 3:**
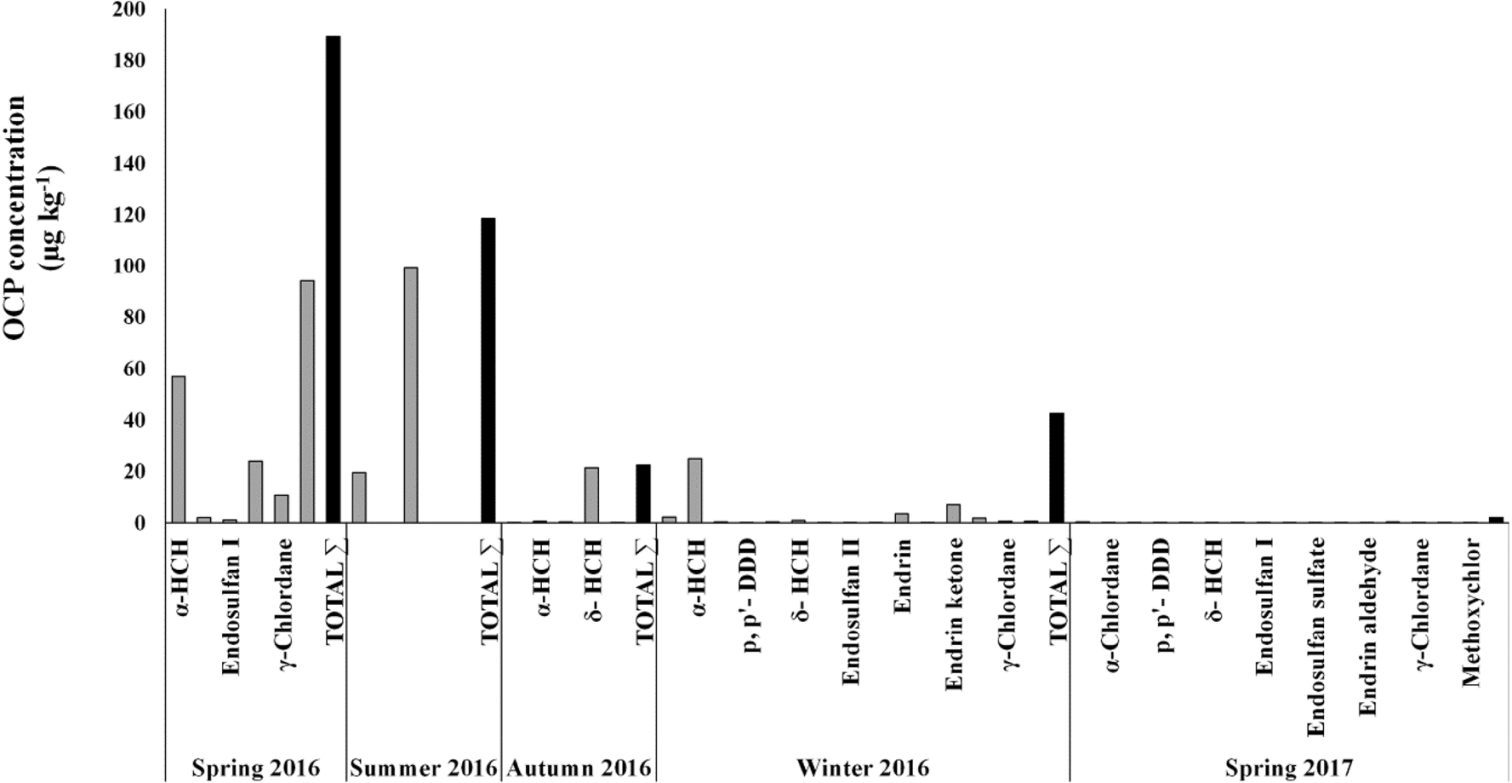
Seasonal concentrations of OCP in the *Sphoeroides* spp. fillet from Navachiste coastal lagoon, Mexico.

The presence of OCPs in *Sphoeroides* spp. tissue was detected in the five collection periods. The spring and summer of 2016 were the seasons with the highest concentration of OCP, with the highest concentrations of methoxychlor, α‒HCH endosulfan sulfate, and γ‒chlordane (94.29, 56.95, 23.94, and 10.80 µg kg^−1^ dw, respectively), whereas, in the summer, the highest concentrations corresponded to β‒HCH and γ‒chlordane (99.18 and 19.48 µg kg^−1^ dw, respectively) (Fig. 3).

### TM risk of *Sphoeroides* spp.

The EDI values for the consumption of *Sphoeroides* spp. and their TM content indicate that most of the TM analyzed, except for Pb, do not exceed the EDI values. Pb exceeded the maximum recommended daily limit by 28.8-times at a rate of 32 g day^−1^ of *Sphoeroides* spp. fillet, representing a potential risk of long-term non-carcinogenic effects due to its consumption (Table 2).

**Table 2:** Concentration, estimated daily intake (EDI), target hazard quotient (THQ), and hazard index (HI) for trace metals analyzed in the edible fillet of *Sphoeroides* spp. from Navachiste, Mexico.

Metal	Concentration (mg kg^−1^ dry wt)	EDI (mg kg^−1^ dry wt)	THQ	ILCR
Cd	1.45	2.38	0.06	9.5 × 10^–2^
Cu	5.06	37.82	0.05	2.6 × 10^–1^
Fe	80.52	2.88	0.02	4.0 × 10^–1^
Mn	6.13	3.79	0.19	7.6 × 10^–2^
Ni	8.06	89.03	0.30	6.8 × 10^–4^
Pb	18.42	0.68	0.68	2.7 × 10^–1^
Zn	189.55	8.65	2.16	3.5 × 10^–2^
			HI = 2.84	

### OCP risk in *Sphoeroides* spp.

The non-carcinogenic and carcinogenic risk values were obtained for each OCP, considering a probability of 5/100,000 individuals having symptoms during a lifetime (Table 4). The average concentration of each OCP did not exceed the RfD values. The calculated HQ values < 100 and THQ = 55.2 do not imply a risk of having symptoms of non-carcinogenic diseases in the mid- or long-term after consuming this species of the NAV.

## Discussion

### TM concentration in *Sphoeroides* spp.

The concentration of Cd (1.45 mg kg^−1^ dw) below other fish species could be related to *Sphoeroides* spp. feeding habits and its place in the trophic web (Table 3). The top predatory fish in the open sea are found in most cases in the upper levels of the food web^59^, depending on whether their feeding habit is filtering or detritivorous^35,60^, or demersal or pelagic^61^. These characteristics make these species suitable for larger biomagnifying and bioaccumulating of trace metals than *Sphoeroides* spp., which an omnivorous species located at the mid-level of the food web, its diet includes fish, crustaceans, and mollusks.

Even though Cu is an essential nutrient for the synthesis of proteins and functioning of enzymes, its consumption in excess presents adverse effects on human health^10^. In the present study, Cu showed higher concentrations (5.06 mg kg^−1^ dw) compared to other carnivorous fishes^62,63^ (Table 3); this could be attributed to the level of TM pollution in NAV caused by human activities^13^, and the position of carnivorous fishes in the food web due to biomagnification^52,64^.

Ni is not essential for human health, but it is toxic above 0.5 mg kg^−1^. The latest reports indicate that the presence of Ni in marine organisms is due to anthropogenic or natural sources, but that in areas with high oil industrial activity the values rise^65^, which may represent risks to human health. However, in NAV there is no oil industry like the one found in the Gulf of Mexico and, as in previous studies^66^, its presence could be due to a lithogenic origin. The Ni concentration (8.06 mg kg^−1^ dw) of the edible tissue of *Sphoeroides* spp. were higher to those reported in recent studies on other marine species of the region^35,60,67–69^ (Table 3). However, the concentration of Ni in predatory fishes has been reported to be slightly higher than those in herbivorous and omnivorous species^66^.

The highest concentration of Zn (189.55 mg kg^−1^ dw) could be a response to lithogenic or anthropogenic sources^70^, such as the increased number of boats due to tourism and artisanal fisheries activities^13^, the effluents from the thermal power plant^71^, and the chemical fertilizers wastes from the neighbored agricultural valley of Guasave. All together could increase the concentration, bioavailability and bioaccumulation of Zn in the environment^72,73^, increasing it in the tissues of aquatic organisms^72,74^; due to its essential micronutrient role as a component of enzymes and oxides, it is automatically adsorbed by the body^75^.

Iron is an important metal for life, essential as a component of proteins, such as hemoglobin, and of muscle tissue^76^. The origin of its high concentration in the analyzed fillet could be attributed to the presence and erosion of this element from the earth crust in the region, or by the untreated sewage discharges from municipal and rural populations to the lagoon^35,60^. This Fe concentration in the present study (80.52 mg kg^−1^ dw) was similar to that detected in *Atherina hepsetus* (78 mg kg^−1^)^77^, higher than in farmed snapper species (5.103–19.985 mg kg^−1^)^78^ (Table 3). These differences can be attributed to the detritivorous feeding habits from sediments rich in Fe and the metabolic differences among species.

In the case of Pb, its concentration (18.42 mg kg^−1^ dw) in *Sphoeroides* spp. was higher than in other species of carnivorous fishes^62,63^ (Table 3), and could be attributed to Pb in sediments and water due to agricultural residues^74^. The latter could reflect the impact of the economic development in the last decade and has been related to the increased amounts of vehicles and traffic and the use of leaded gasoline or diesel or to the mining residues that could be carried by rivers^79,80^.

Mn is considered a micronutrient, enzyme activator, and main component in mitochondrial enzymes such as superoxide dismutase and pyruvate carboxylase^11^, but, above certain concentrations, it generates damage at the genetic, enzymatic, or neurological level^81,82^. The Mn concentration detected in the *Sphoeroides* spp. (6.13 mg kg^−1^ dw) was above the MRL (0.140)^44,83^. In relation to other species, Mn showed a discrepancy with other marine fish species (Table 3). Due to the critical role of Mn in fish metabolism, it is immediately absorbed due to its involvement in gill metallothionein levels, oxidative protein damage in liver and muscle, and gill activity of superoxide dismutase; it is influenced by the trophic level and feeding habit of the species^84^.

**Table 3:** Trace metal concentrations (mg kg^−1^ dry wt) in marine fishes *Sphoeroides* spp. and other regions in the world.

Species	Cu	Fe	Mn	Ni	Cd	Zn	Pb
*Sphoeroides* spp.^*†*^	5.06	80.52	6.13	8.06	1.45	189.55	18.42
*Apocryptes bato*^*a*^	46.98	151.17	–	–	–	101.38	0.65
*Arius arius*^*f*^	6.51	112.3	3.65	–	0.02	43.53	0.14
*Arius maculetus*^*f*^	1.75	107.3	2.24	–	0.49	55.3	0.26
*Carangodiae* sp.^*f*^	5.8	91.5	1.7	–	0.42	52.9	0.12
*Carcharhinus leucas*^*e*^	13.3	–	0.4	4.1	0.6	71.0	2.4
*Coilia dussumieri*^*f*^	2.24	105.8	7.75	–	0.5	38.81	-
*Coilia dussumieri*^*f*^	5.59	207.2	3.87	–	0.14	54.83	0.17
*Cynoglossus senegalensis*^*c*^	–	–	314	16.6	0.4	113	–
*Dentrophysa russelli*^*f*^	1.65	78.05	2.12	–	0.08	38.69	0.13
*Harpadon nehereus*^*a*^	35.4	148.82	–	–	–	106.72	0.2
*Johnius elongatus*^*f*^	2.15	240.5	4.47	–	0.57	41.45	0.22
*Johnius macropterus*^*f*^	0.87	74.93	2.39	–	0.04	20.3	0.11
*Lates calcarifer*^*a*^	31.44	135.58	–	–	–	103.72	0.44
*Liza macrojepis*^*f*^	1.62	68.93	1.6	–	0.02	26.21	0.04
*Lutjanus johni*^*f*^	1.88	62.31	2.08	–	0.07	25.55	–
*Mystus gulio*^*a*^	46.57	150.81	–	–	–	129.33	0.65
*Otolithes ruber*^*e*^	12.4	–	1.55	–	0.3	16.9	1.6
*Otolothoides pama*^*a*^	30.29	177.46	–	–	–	107.22	0.38
*Pegusa lascaris*^*b*^	0.16	–	–	–	0.005	0.2	0.11
*Plotosus limbatcus*^*f*^	1.27	51.58	1.48	–	0.08	14.38	0.09
*Polynemus paradiseus*^*a*^	33.95	188.15	–	–	–	101.38	0.48
*Polynemus tetraductylus*^*f*^	1.84	32.11	1.17	–	0.47	31.2	0.01
*Psettodes erumei*^*e*^	9.9	–	3.6	–	0.4	36.6	2.6
*Sardine longiceps*^*d*^	3.61	18.71	0.38	–	0.27	15.87	0.24
*Scatophagus argus*	2.41	84.99	4.99	–	0.11	34.53	0.13
*Scomberomorus lineolatus*^*e*^	8.50	–	0.70	1.20	0.20	20.2	1.70
*Sparus aurata*^*b*^	0.13	–	–	–	0.002	0.2	0.05
*Synaptura commersonnii*^*d*^	4.59	78.48	3.48	–	0.47	26.27	0.89
*Tautoga onitis*^*b*^	0.296				0.002	3	0.007
*Tetraden* sp.^*f*^	2.4	101.2	1.96	–	0.11	30.34	–
*Therapon jarbua*^*f*^	2.54	70.63	2.9	–	0.04	57.72	0.14
*Thryssa mystax*^*f*^	1.55	53.87	5.65	–	0.03	60.75	0.16
*Thryssa hamiltonii*^*f*^	1.98	69.26	7.27	–	0.08	53.11	0.02
*Trachurus mediterraneus*^*b*^	0.23	––	–	–	0.03	0.3	0.05
*Trichiurus lepturus*^*f*^	2.11	141	6.34	–	0.12	42.34	0.04
*Trichiurus lepturus*^*e*^	9.0	–	3.4	–	0.51	25.3	0.6
*Trypauchen* sp.^*f*^	1.16	40.74	1.4	–	0.1	12.77	0.24

^a^Hossain et al., 2022;^b^Karayakar et al., 2022;^c^Joseph et al. 2022;^d^Adani et al. 2022;^e^Anandkumar et al. 2018;^f^Velusamy et al., 2014;^*†*^This study.

The bioavailability of TM depends mainly on the sediment's physicochemical characteristics, chemical fractions, and pH, most of them affect their bioavailability^85^. However, the chemical form in which they are found and the anthropogenic contributions increase their basal concentration in a specific site. In the case of NAV, it is adjacent to more than 150,000 ha of intensive agricultural activities (> 160,000 ha). The presence of Cd, Zn, and Cu coincides with the use of fertilizers and pesticides by this agricultural area^86^, shrimp culture, and domestic sludge, which influence the concentration in the seawater and sediments of the NAV region^60^. The concentrations of Cd, Cu, and Ni revealed the significant relationship previously reported with trophic levels. Benthic invertebrates have shown a species-specific accumulation of these TM in the food web rather than biomagnification^52^, and it has been reported that bioaccumulation of TM depends on fish feeding habits and the inhabited region^54^. The TM concentrations in *Sphoeroides* spp. are higher than those previously reported in marine fishes^83,87,88^. This higher concentration depends on various factors, the feeding habit of *Sphoeroides* spp. as carnivorous, the enrichment factor in the sediments, the continental crust contribution, and the grade and source of anthropogenic pollution^13,28,35,89^. Compared with recent reports on the concentration of TM in the muscle of other marine fish species, the concentrations of Cd, Ni, Zn, and Pb in the *Sphoeroides* spp. fillet were higher. Lower concentrations of Cu and Fe have been reported in A*pocryptes bato*, *Harpadon nehereus*, *Polynemus paradiseus,* and *Otolothoides pama* from coastal areas around the mouth of the Meghna River in Bangladesh; a lower concentration of Mn has been reported in *Cepola macrophthalma* in Karatas, Turkey^90,91^ (Table 3). The concentration of TM depends on the degree and sources of anthropogenic contamination present in the areas and the feeding habits of the species^62^. As explained above, Navachiste is constantly impacted by effluents from intensive irrigation residues from the neighboring agricultural valley, and *Sphoerpoides* spp. is a pelagic and benthic carnivorous species. Therefore, its location in the food web allows it to bioaccumulate biomagnified TM. The same occurs with the species reported for Bangladesh. Although the area where those species were caught are defined as marine species^90^, they possibly were captured from coastal areas impacted by human activities.

### OCP concentrations in *Sphoeroides* spp.

The high ratios of *p, p'*‒ DDE (0.52) and *p, p'*‒DDD (0.48), and the absence of *p, p'*‒ DDT suggest that there have been no recent applications of *p, p'*‒DDT in the area. DDE is the most persistent metabolite of *p, p'*‒DDT in the environment that can last up to 10 years available in the environment^92^. The presence of non-detected *p, p'*‒DDT concentration in the edible tissue of *Sphoeroides* spp. suggests the persistence of residues from the 60's to the '90 s in the sediments of NAV. During that time, *p, p'*‒DDT was an insecticide used intensively to control insects in crops^93^. This persistence can be corroborated by the present detected low concentrations of *p, p'*‒ DDT in *Sphoeroides* spp. previously detected in fishes from the same study area^17,18^ Currently, in Mexico, technical *p, p'*‒ DDT is an OCP commercially banned but of restricted use exclusively by the Mexican Ministry of Health for the control of vectors of infectious diseases such as the mosquito that mainly transmits dengue.

The high aldrin and lower endrin and dieldrin concentrations could be due to the recent use of the three pesticides on agricultural crops^45,94^. In the present study, the higher ratios for dieldrin (0.43) and endrin (0.47) than for aldrin (0.1) indicate historical contamination. In this case, most of the dieldrin available in the environment could be originated from the oxidation of aldrin, as previously reported^95^; in Mexico, as stated above it is actually prohibited, but it was very popular in the past as an insecticide in agriculture^93,96^.

Technical grade HCH is a mixture of isomers of this molecule, *α*‒HCH (60–70%), *β*‒HCH (5–12%), *γ*‒HCH (10–15%), *δ*‒HCH (6- 10%), the commercial lindane product consists of 99% γ‒HCH, and the presence of all HCH isomers and the ratio of *γ*‒HCH to the rest indicates historical contamination from the use of lindane and technical HCH^40,97,98^.

Technical chlordane has a restricted use as a termiticide, and it is not prohibited in Mexico. The ratios of the isomers, *α*‒ chlordane (0.0007), *γ*‒chlordane (0.98), heptachlor (0.007), and heptachlor epoxide (0.003), and the low frequency of the last two in the samples imply a historical use and might be attributed to atmospheric transport or their runoff in the last decades from the neighboring agricultural area.

The degradation product of technical endosulfan to endosulfan sulfate, could result as a product of the metabolism of some fungi such as *Trametes versicolor* and *Pleurotus ostreatus*^99^; and which was detected with an average concentration of 4.93 µg kg^−1^ and a frequency of 11.63% of the samples. In this way, it is plausible that the residual products of endosulfan and its isomers from the neighboring agricultural valley indicate endosulfan's recent use due to its low persistence between 30 and 150 days^100^, resulting in the bioaccumulation by *Sphoeroides* spp.

Methoxychlor is used as a larvicide in crops and can persist in the environment for up to 6 months^101^, and the detection in the fillet of *Sphoeroides* spp. would imply a recent use in the agricultural area of the Valle de Guasave even its use is restricted to the exclusive use in seed treatment for sowing in crops of rice, oats, barley, peas, beans, corn, sorghum^102^.

The bioavailability of OCPs has been correlated with the yearly seasons, their concentrations have been reported to be higher in the seasons after the rainy season or in the dry season^103,104^. In this study, the same characteristics were found, the highest concentrations occurred in the spring after the intensive agricultural irrigation season in the zone^35^, and OCP become bioaccumulated by marine organisms^105^.

### TM risk of *Sphoeroides* spp.

The THQ for each metal, except for Pb (THQ = 2.16), was less than 1. The Pb value indicates a high possibility of non-carcinogenic effects in the mid-term due to the consumption of *Spheroides* spp. from NAV at 32.88 g day^−1^. This result regarding the toxic implications of Pb in the edible tissues of marine fish have been previously reported^61^. However, if the consumption ratio of *Sphoeroides* spp. fillet increases to a rate of 120 g day^−1^ on average (equivalent to three "tacos"), the chances of presenting symptoms due to non-carcinogenic effects increase substantially for Pb (THQ = 7.89) and other TMs such as Cd (2.49) and Zn (1.08).

Regarding the carcinogenicity risk, the ILCR values were greater than 1 × 10^–6^ (lifetime cancer risk probability), and the risk is not significant if the ILCR value is lower than 1 × 10^–6^. In the present study, all ILCR values of the analyzed TM were above 1 × 10^–6^, indicating a high risk for cancer development in the long-term (Table 2)^106^. Nevertheless, the cooking procedures could reduce the bioaccessibility and concentrations of the TM in the edible tissue of marine fish^107,108^.

It has been previously reported how pollutant residues inputs are deposited in the sediments of the coastal lagoons close to agricultural drains^80^, such as TM residues, which have been reported in edible tissues of the marine species and sediments inhabiting those ecosystems^17,18,20,35,69,109^. The discrepancies in the levels of heavy metals among the different studies could be due to the difference in fish metabolism, metal bioactivity, species of fishes, and trophic levels^55,59^, type of contaminants, geographical location^110^, capture season, fish size, fish age^52,55^, and the detainment period of metals in water^111,112^.

### OCP risk in *Sphoeroides* spp.

Regarding the carcinogenic risk (CRLim), only aldrin, p, p′‒ DDD, p, p′‒ DDE, dieldrin, endosulfan, heptachlor, heptachlor epoxide, α‒HCH, β‒HCH, and γ‒HCH CR_Lim_ showed values that represent a high probability for developing cancer in the long-term (Table 4). These values were higher than those reported in edible muscle in fishwhich CR_Lim_ could allow eating higher portions of them before reaching a potential health risk^40,113^. In the present study, the CR_Lim_ of some OCPs was remarkably lower than the meal size analyzed here (32.88 g), implying that the consumption of the edible fillet of *Sphoeroides* spp. over a long time could be a potential cause of cancer at this meal portion. Nevertheless, the amount of OCP considered here was in raw fish tissue, and factors such as the bioavailability of pesticides in the tissue, the possibility that the ingested OCPs are totally or partially excreted, or the amount lost during the cooking process of the fillet may alter the concentration of OCP^40^. As reported, the cooking process used (microwaving, roasting. and boiling) will reduce the concentrations of OCP in the edible tissue of marine fish^114^.

**Table 4:** OCP concentration (mg kg^−1^), oral slope factor (OSF), reference dose (RfD), estimated daily intake (EDI), target hazard quotient (THQ), hazard quotient (HQ), and cancer risk limit (CR_Lim_) in *Sphoeroides* spp. of the Navachiste Lagoon System.

OCP	Concentration	OSF	RFD	EDI	HQ	CR_Lim_	THQ 55.2
Aldrin	0.00062	17	0.0003	0.00028	1.257487	0.0053	
p, p′‒DDD	0.0003	0.24	ND	0.00014		2.7812	
p, p′‒DDE	0.00012	ND	ND	0.00005		1.7769	
Dieldrin	0.00014	0.34	ND	0.00006	0.123454	0.0342	
Endosulfan	0.00022	ND	0.006	0.0001	0.001957		
Endosulfan II	0.0002	ND	ND	0.00009			
Endosulfan sulfate	0.0027	ND	ND	0.0012			
Endrin	0.002	ND	ND	0.0009	0.347707		
Endrin aldehyde	0.00005	ND	ND	0.00002			
Endrin ketone	0.0025	ND	0.0003	0.0011			
Heptachlor	0.00011	4.50	0.00005	0.00005	0.015652	0.0960	
Heptachlor epoxide	0.0011	9.10	0.00001	0.0005	0.269983	0.1059	
Methoxychlor	0.0052	ND	0.005	0.0024	0.057705		
α‒Chlordane	0.00009	ND	ND	0.00004			
γ‒Chlordane	0.022	ND	ND	0.0099			
α‒HCH	0.025	6.3	0.008	0.0113	0.144997	0.0005	
β‒HCH	0.0035	1.8	ND	0.0017		0.0113	
δ‒HCH	40.005	0.24	ND	0.0022			
γ‒HCH	0.0017	ND	0.0003	0.0008	0.268175	0.0053	

ND, below detection limits.

## Conclusions

The health risk posed by the TM and OCPs concentration in the edible tissue of *Sphoeroides* spp from the NAV in the southwestern part of the Gulf of California was evaluated. The results of non-carcinogenic and carcinogenic risks revealed that the Pb value pose a high possibility of inducing non-carcinogenic effects in the mid-term. However, if the consumption ratio increases up to 120 g day^−1^ of edible tissue (equivalent to three “tacos” per day), the non-carcinogenic effects symptoms increase substantially for Pb and become potential for Cd and Zn. The evaluated OCP carcinogenic risks highlight that the ILCR values were more significant for a lifetime cancer risk probability, and the TM analyzed here were above minimum ILCR (> 1 × 10^–6^), indicating a high-risk potential for the development of cancer in the long-term. OCP´s did not exceed the RfD values, and the HQ values did not imply a risk to present non-carcinogenic diseases in the mid- or long-term. The CR_Lim_ of aldrin, *p, p'*‒ DDD, *p, p'*‒ DDE, dieldrin, endosulfan, heptachlor, heptachlor epoxide, α‒HCH, β‒HCH, and γ‒HCH CR_Lim_ had values that represent a high probability for developing cancer in the long-term. It is evident that if local communities consume the fillet of the *Sphoeroides* spp. in portions above 120 g day^−1^ it could represent a carcinogenic and non-carcinogenic risks in the mid and long term. Nevertheless, if the fillet is cooked, mainly boiling, frying or steaming, it could reduce the bioaccessible OCP and TM fractions. Intensive anthropogenic activities constantly dispose of their residues, after irrigation or water exchange, directly into the NAV through discharge channels, which is evidenced by the pesticides and chemical residues pollution recorded in the NAV sediments.

## Supplementary Information

Supplementary Table 1.

Supplementary Table 2.

Supplementary Table 3.

## Data Availability

The datasets used and/or analyzed during the current study are available from the corresponding author on reasonable request.
