# Supplementary material for: Health risk of consuming Sphoeroides spp. from the Navachiste Lagoon complex due to its trace metals and organochlorine pesticides content
Source: Sci Rep. 2022 Nov 1;12:18393. doi: 10.1038/s41598-022-22757-1 (PMC9626642; doi:10.1038/s41598-022-22757-1)
Supplement: Supplementary file 1 — Supplementary Table 1. [file 41598_2022_22757_MOESM1_ESM.docx]

**Supplementary Table 1.** Coordinates dataset of sample collection sites of specimens of *Sphoeroides* spp. to determination of its trace metal and organochlorines pesticides content in the edible tissue in Navachiste coastal lagoon, Mexico.
