# Supplementary material for: Health risk of consuming Sphoeroides spp. from the Navachiste Lagoon complex due to its trace metals and organochlorine pesticides content
Source: Sci Rep. 2022 Nov 1;12:18393. doi: 10.1038/s41598-022-22757-1 (PMC9626642; doi:10.1038/s41598-022-22757-1)
Supplement: Supplementary file 2 — Supplementary Table 2. [file 41598_2022_22757_MOESM2_ESM.docx]

| **Supplementary Table 2.** Reference materials and recovery range to determine the quality of analytical methods. | | |
| --- | --- | --- |
| **Trace metal** | **Certified reference material** | **Recovery range** |
| Pb | PACS | 91.75% |
| Zn | TORT | 79.87% |
|  | PACS | 79.23% |
| Cd | TORT | 92.37% |
| Ni | TORT | 89.61% |
|  | PACS | 104.91% |
|  | MESS | 101.62% |
| Mn | 1570a | 85.26% |
| Fe | 1560 | 77.10% |
|  | TORT | 96.93% |
| Cu | 1560 | 89.34 |
|  | 1570 | 94.31 |
|  | TORT | 89.60 |
