# Supplementary material for: Health risk of consuming Sphoeroides spp. from the Navachiste Lagoon complex due to its trace metals and organochlorine pesticides content
Source: Sci Rep. 2022 Nov 1;12:18393. doi: 10.1038/s41598-022-22757-1 (PMC9626642; doi:10.1038/s41598-022-22757-1)
Supplement: Supplementary file 3 — Supplementary Table 3. [file 41598_2022_22757_MOESM3_ESM.docx]

| **Supplementary Table 3**. Detection limits, mean recovery, sample recovery range, and coefficient of variation. | | | | | |
| --- | --- | --- | --- | --- | --- |
| OCP | Linearity  R^2^ (N=12) | Detection limit  (ugkg^-1^) | Recovery range  (Mean ± SD) | | Variance coefficient (%) |
| Aldrin | 0.986 | 0.003 | 0.736 | 0.48 | 153.305 |
| Dieldrin | 0.92 | 0.007 | 0.088 | 0.078 | 111.867 |
| Endosulfan I | 0.913 | 0.007 | 0.164 | 0.122 | 135.17 |
| Endosulfan II | 0.978 | 0.004 | 0.331 | 0.178 | 185.788 |
| Endosulfan sulfate | 0.942 | 0.002 | 0.088 | 0.064 | 136.324 |
| Endrin | 0.989 | 0.007 | 0.237 | 0.144 | 164.553 |
| Endrin aldehyde | 0.976 | 0.004 | 0.028 | 0.024 | 113.842 |
| Endrin ketone | 0.996 | 0.002 | 0.218 | 0.142 | 153.357 |
| Heptachlor | 0.956 | 0.007 | 0.909 | 0.672 | 135.325 |
| Heptachlor epoxide | 0.913 | 0.007 | 0.349 | 0.316 | 110.161 |
| Metoxychlor | 0.982 | 0.002 | 0.298 | 0.199 | 150.069 |
| *p, p'‒*DDD | 0.98 | 0.007 | 0.256 | 0.141 | 181.08 |
| *p, p'‒*DDE | 0.91 | 0.007 | 0.13 | 0.107 | 121.997 |
| *p, p'‒*DDT | 0.988 | 0.002 | 0.401 | 0.222 | 180.404 |
| α‒Chlordane | 0.923 | 0.006 | 0.198 | 0.156 | 126.762 |
| γ‒Chlordane | 0.913 | 0 | 0.534 | 0.487 | 109.638 |
| α‒HCH | 0.973 | 0.008 | 0.536 | 0.429 | 124.914 |
| β‒HCH | 0.991 | 0.003 | 0.384 | 0.307 | 124.906 |
| δ‒HCH | 0.991 | 0 | 0.03 | 0.023 | 128.71 |
| γ‒HCH | 0.993 | 0.003 | 1.175 | 0.833 | 141.158 |
